# Two New Kindreds with Complete Factor D Deficiency

**DOI:** 10.1002/eji.202451536

**Published:** 2025-03-12

**Authors:** Mathilde Puel, Kenza Rwayane, Paula Vieira Martins, Marwa Chbihi, Frédéric Rieux‐Laucat, Jérémie Rosain, Eric Jeziorski, Bertrand Boisson, Jean‐Laurent Casanova, Véronique Frémeaux‐Bacchi, Carine El Sissy

**Affiliations:** ^1^ Department of Immunology Assistance Publique‐ Hôpitaux de Paris (AP‐HP) Georges Pompidou European Hospital Paris France; ^2^ Pediatric Immunology‐Hematology and Rheumatology Unit, Assistance Publique ‐ Hôpitaux de Paris (APHP) Necker‐Enfants Malades Hospital Paris France; ^3^ Laboratory of Immunogenetics of Pediatric Autoimmune Diseases Imagine Institute INSERM UMR 1163 Paris France; ^4^ Laboratory of Human Genetics of Infectious Diseases, Necker Branch INSERM U1163 Necker Hospital for Sick Children, EU Paris France; ^5^ Study Center for Primary Immunodeficiencies Assistance Publique Hôpitaux de Paris (AP‐HP) Necker Hospital for Sick Children, EU Paris France; ^6^ St.Giles Laboratory of Human Genetics of Infectious Diseases Rockefeller Branch Rockefeller University New York USA; ^7^ University of Paris Cité Paris France; ^8^ Pathogenesis and Control of Chronic Infections INSERM U1058 Montpellier UHC University of Montpellier Montpellier France; ^9^ Department of General Pediatrics Infectiology, and Clinical Immunology Department of Emergency Post‐Emergency Department University Hospital of Montpellier Montpellier France; ^10^ Inflammation, Complement and Cancer Team Cordeliers Research Center Institut National de la Santé et de la Recherche Médicale (INSERM) Unité Mixte de Recherche UMRS1138 Paris France; ^11^ COMET « Complement Expertise and Therapeutics » Fédération Hospitalo‐Universitaire Paris France

## Abstract

Inborn deficiencies of the alternative pathway (AP) of the complement system have been associated with life‐threatening infections, mainly by encapsulated bacteria. Complete factor D (FD) deficiencies have been reported in only seven families in the literature. We report two new cases of biochemically and genetically confirmed complete FD deficiency, including the first in a Down syndrome patient. The index cases respectively suffered from severe *H. influenza* and *N. meningitidis* infections. Their FD activity was undetectable but was restored by adding recombinant human FD. FD levels were undetectable in the plasma of both patients using ELISA. Genetic analysis of the *CFD* gene identified a homozygous missense variant p.M40R in one patient, and compound heterozygous variants—a nonsense mutation p.Cys148* and a splice site variant c.212+2T>G—in the other. Patients with Down syndrome are more susceptible to infections, but this case highlights the importance of investigating the complement system, particularly the AP, even in those with Down syndrome or other secondary immune deficiencies. A familial study should follow if a congenital deficiency is found. The natural history of patients with inherited complete FD deficiency underscores the necessity of preventive measures against encapsulated bacteria for those receiving therapeutic MASP‐3 or FD inhibitors.

## Graphical abstract

The complement system plays a crucial role in immune defense, and factor D deficiency is a rare immunodeficiency associated with life‐threatening bacterial infections. We report two new cases of complete factor D deficiency, including the first described in a Down syndrome patient, highlighting the need to investigate complement deficiencies in patients with secondary immunosuppression. Given the increased susceptibility to infections, our findings underscore the importance of preventive measures for patients receiving therapeutic MASP‐3 or factor D inhibitors, which mimic the effects of inherited factor D deficiency.

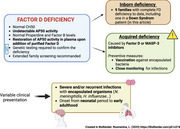

**Abbreviations**
AP: alternative pathway of the complement system
AP50: functional complement activity assay of the alternative pathway
CH50: functional complement activity assay of the classical pathway
CP: classical pathway of the complement system
DS: Down syndrome
FB: complement factor B (CFB complement FB gene)
FD: complement factor D (CFD complement FD gene)
ICU: intensive care unit
PNH: paroxysmal noctural hemoglobunria

## 1 Introduction

The complement system is composed of more than 35 proteins and is an essential actor in the innate and adaptive immune response to pathogens, especially encapsulated bacteria [1]. It is also involved in homeostasis and the immunomodulation of the adaptive immune response, meaning that defects associated with this system are either associated with increased sensibility to pathogens or inflammation and autoimmunity [2]. Activation of the classical (CP) or the alternative (AP) pathway results in the cleavage of C3, the central component of the complement system. In the alternative pathway, the spontaneous hydrolysis product C3(H_2_O) complexes with complement factor B (FB). In this complex, FB is cleaved by complement factor D (FD) to its active form, resulting in the C3(H_2_O)Bb complex, which serves as an unstable fluid‐phase C3 convertase that cleaves C3 into C3a and C3b. In the presence of a pathogen, C3b will covalently attach to the surface and bind FB which will be cleaved by FD to Bb and Ba forming the alternative pathway C3 convertase (C3bBb). Through this mechanism, activation of the AP can be rapidly amplified (“amplification loop”). An increasing number of C3b will bind near the C3 convertase to generate the AP C5 convertase, C3bBb3b. This last enzyme binds and cleaves C5, with the subsequent generation of C5a and C5b. Interaction of C5b with the terminal components of the complement system C6, C7, C8, and C9 results in the formation of the “membrane attack complex.”

Complement FD has an essential role in the initiation and propagation of the AP activation which contributes significantly to responses against bacterial infection. Human FD is a 24 kDa serine protease [3] that circulates in blood at approximately 2 to 5 µg/mL. Unusual among complement proteins, FD is synthesized by adipocytes and macrophages [4]. Complete or near‐complete FD deficiency has been described so far in only 14 individuals from seven kindreds [2, 5, 6, 7, 8, 9]. Complete FD deficiency is associated with an increased susceptibility to invasive infections, particularly those caused by encapsulated organisms such as *Neisseria meningitides*. The consequences for complete FD‐deficient patients are as severe as those observed in individuals with deficiencies in terminal or other alternative pathway components such as properdin.

Although hereditary FD deficiency is extremely rare, with only seven kindreds reported in the literature, the prevalence of acquired FD deficiency is expected to rise with the advent of new FD inhibitors. This article aims to underscore the associated risks and raise awareness of this emerging concern.

Here, we report two new unrelated kindreds with FD deficiency and provide a literature review of previously reported cases of complete FD deficiency.

## 2 Patients, Material, and Methods

### 2.1 Complement System Exploration

Functional complement activity assay of the classical (CH50) and alternative pathway (AP50) hemolytic activities were determined according to standard procedures [10]. CH50 and AP50 are based on the lysis of Ab‐sensitized sheep and rabbit erythrocytes, respectively, and results were rendered in the percentage of activation compared with a normal pool [11]. To avoid *in vitro* complement activation, blood was drawn directly into EDTA‐containing tubes. Plasma concentrations of C3 and C4 were measured by nephelometry (Siemens, Inc., Newark, DE) as previously described [2].

To evaluate the functional activity of complement FB and FD, a hemolytic test similar to the AP50 test was conducted with recombinant FB or FD supplementation. Patient plasma was mixed with rabbit erythrocytes after adding a complement activator at physiological concentrations of either purified recombinant FB or FD (CompTech, Tyler, TX). If the addition of a recombinant complement factor restored the ability of the patient's plasma to hemolyze rabbit erythrocytes, the patient was considered deficient in that complement factor.

FD concentration in patient plasma was measured by ELISA (complement factor D Human ELISA kit, ThermoFischer Scientific, South San Francisco, CA) according to manufacturer recommendations. A pool of healthy donors served as control.

### 2.2 Genomic DNA sequencing

Complement FD deficiency was confirmed by genetic testing. Genomic DNA was extracted and purified from whole blood using standard procedures. Sequencing of the *CFD* gene was performed by NGS as previously described [2].

### 2.3 Ethics

Patients or legal tutors of minor patients gave informed written consent prior to the functional and genetics exploration needed and for the familial studies.

## 3 Results

### 3.1 Patient's Details

We studied two unrelated patients who presented with life‐threatening infections caused by encapsulated bacteria, *Haemophilus influenzae* and *N. meningitidis*, respectively.

#### 3.1.1 Patient 1

**FIGURE 1:**
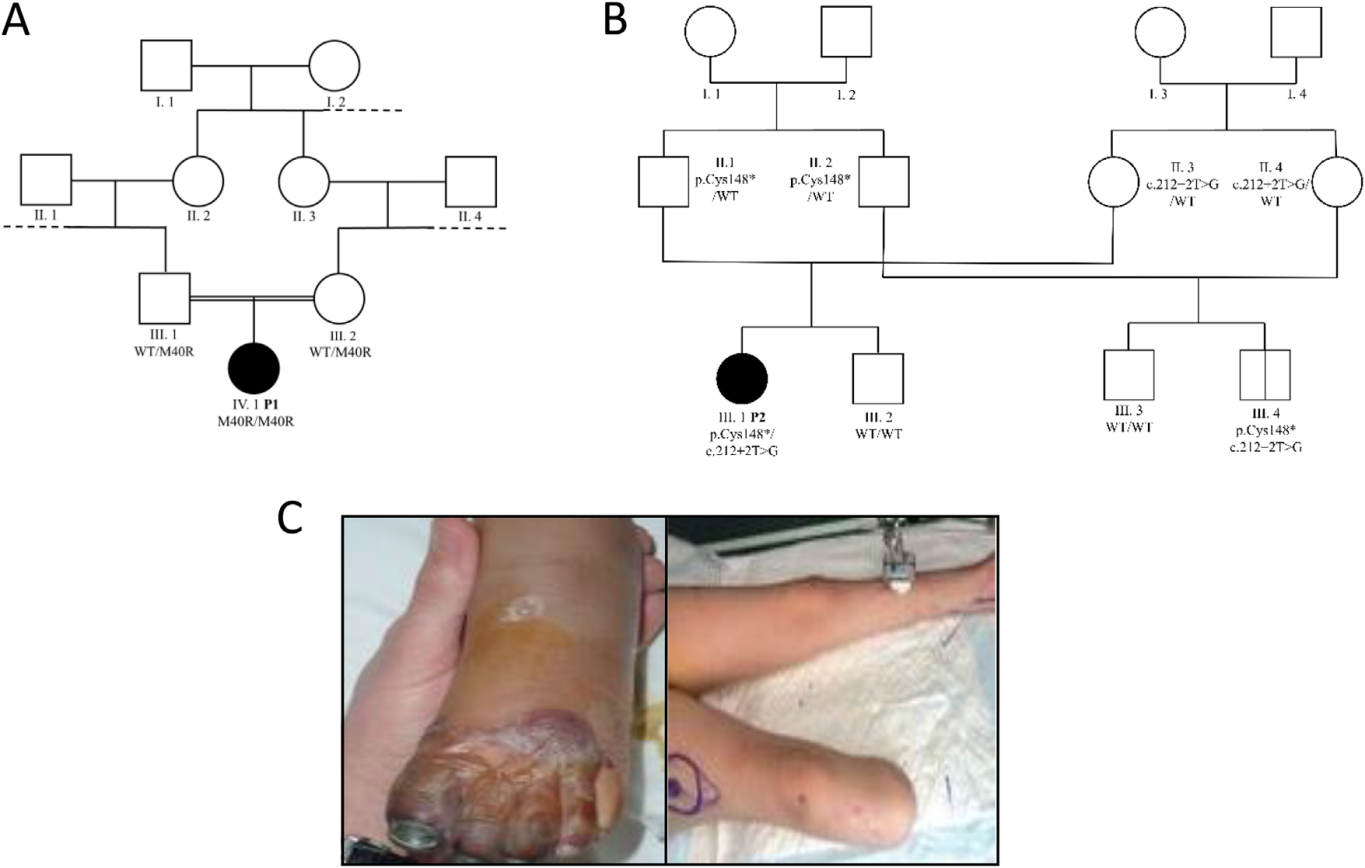
Familial and clinical descriptions of patients P1 and P2. (A) Genealogical tree of family A (patient P1); (B) genealogical tree of family B (patient P2); (C) necrotic lesions of P2.

Patient P1 was a 13‐year‐old girl of Pakistani descent living in France. She was born to consanguineous parents and had no siblings (Figure 1A). Her relatives did not present any medical history. Her past medical history included Down syndrome (DS) but did not reveal recurrent infections in her first 13 years of life. She had received all vaccines, including boosters, recommended for her age according to the French vaccination guidelines, including the meningococcus serogroup C vaccine, the *H. influenzae* type B vaccine, and the 7‐valent pneumococcus vaccine. At 13 years of age, she initially presented with cough, fever, diarrhea, and vomiting. Two days later, she was admitted to the pediatric intensive care unit (ICU) due to severe respiratory distress. She required intubation and was diagnosed with right lung pleuropneumonia. Bronchoalveolar lavage (BAL) fluid and blood cultures were tested positive for *H. influenzae*. BAL fluid tested positive for respiratory syncytial virus and negative for a panel of other respiratory viruses, including SARS‐CoV‐2. She was treated with antibiotics and underwent pleural drainage. In total, she was intubated for 18 days and was discharged from the intensive care unit 39 days after admission. She remained ventilator‐dependent for up to 22 months following the infection and continued to be monitored by the pulmonology department.

#### 3.1.2 Patient 2

Patient P2 (Figure 1B) was an eight‐year‐old girl born to nonconsanguineous parents of French descent. Her relatives had no past medical history. She had no medical history until the age of 8. She had received all vaccines, including boosters, recommended for her age according to the French vaccination guidelines, including the meningococcus serogroup C vaccine, the *H. influenzae* type B vaccine, and the 7‐valent pneumococcus vaccine. At that time, she presented with high fever, vomiting, and asthenia at home. On the morning of her second day of fever, she was evaluated by her general practitioner, who did not observe any meningococcal syndrome or cutaneous lesions. A few hours later, she developed rapidly progressing purpuric lesions and was referred to the ICU. She was diagnosed with septic shock, purpuric lesions, and disseminated intravascular coagulation and was treated with an appropriate course of antibiotics. Microbiological and molecular analyses from whole blood and skin lesions samples tested positive for *N. meningitidis* group B. The cutaneous lesions progressed to severe necrosis of the upper and lower extremities resulting in multiple surgical interventions including debridement, skin grafts, and amputation of the fingers and right leg (Figure 1C). During this prolonged hospital stay, she presented with complications such as nosocomial acute respiratory distress syndrome, which was not documented as a nosocomial infection, and osteitis of the left calcaneum. Subsequently, she was prescribed chemoprophylaxis and received the quadrivalent (ACYW135) *N. meningitidis* vaccine. To date, since this severe episode 8 years ago, she has not experienced any invasive or severe infections, nor required hospitalization due to an infection.

### 3.2 Immunological and Genetic Investigation of the Immune System

**FIGURE 2:**
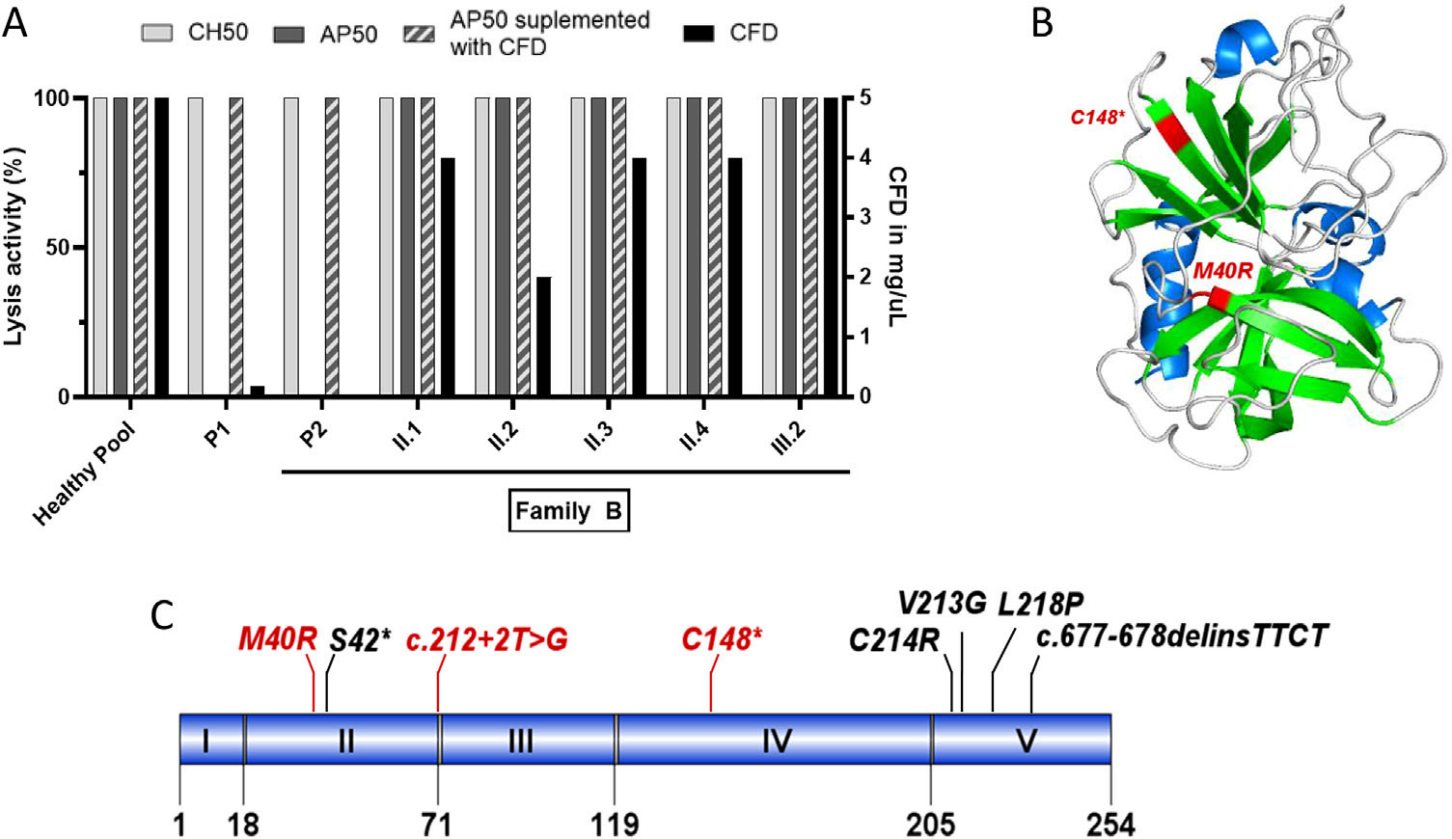
Biochemical and genetical exploration of patients P1 and P2 complement factor D (CFD) deficiency. (A) Complement activity exploration and CFD dosage of P1, her parents, P2, and part of family B. (B) 3D representation of CFD protein with the two nonsense mutations found in P1 and P2 (red). Alpha helix are in blue and beta sheets in green. (C) CFD gene (NM_001928) with variants reported in the article (red) and in the literature (black).

Given the life‐threatening infections that happened, in both kindreds, a complete immunological workup was performed, including a study of the complement system. In both patients, CH50 activity and C3 and C4 levels were within normal ranges but AP50 activity was undetectable (<10% of normal values), suggesting a deficiency in one of the components of the AP. For both patients, the addition of purified FD (CompTech, Tyler, TX) at the physiological concentration of 2 µg/mL restored the AP50 activity (Table 1, Figure 2A). The addition of purified FB (CompTech, Tyler, TX) did not restore AP50 activity (Table 1). Both patients P1 and P2 had undetectable FD levels (<0, 15 µg/mL; Table 1, Figure 2A). The normal FD level was 5 µg/mL, assessed in a pool of healthy individuals (Table 1). We next performed Sanger sequencing of the CFD gene (*CFD*, NM_001928) in both patients. P1, presented with a homozygous nonsynonym substitution c.119T>G (p.Met40Arg) (Figure 2B,C). P2 had two heterozygous variants: a variant impacting the essential splice site (c.212+2T>G) and a stop‐gain variant (c.444C>A, p.Cys148*; Figure 2B,C). Both parents of patient P1 were heterozygous for the c.119T>G mutation. Familial segregation analysis of P2 revealed that the patient had inherited a variant from each parent, one cousin of P2 presented with the same two variants and thus also displayed FD deficiency (Figure 1B). This P2's cousin was a 4‐year‐old boy at the time of diagnosis, with no prior history of invasive infections or significant medical conditions. He had been vaccinated against *N. meningitidis*, *S. pneumoniae*, and *H. influenzae* type B according to French vaccination guidelines. Now 6 years old, he remains asymptomatic. He has been prescribed chemoprophylaxis and received additional vaccinations, including the 20‐valent pneumococcal vaccine and the quadrivalent (ACYW135) meningococcal vaccine. His parents have also been informed of the importance of seeking medical advice promptly if he exhibits any signs of infection.

**TABLE 1:** Complement explorations of two new kindreds with FD deficiency.

Patient	CH50 (70–130%)	AP50 (70–130%)	C3 (660–1250 mg/L)	C4 (93–380 mg/L)	FD µg/mL
P1 (IV.1)	74	<10	1580	307	<0.15
III.1	79	100	1840	330	NA
III.2	100	100	1350	375	NA
P2 (III.1)	60	<10	1210	190	<0.15
III.2	48	100	1330	257	5
III.3	39	100	1030	222	NA
III.4	38	<10	1390	196	NA
II.1	61	100	990	197	3
II.2	101	100	949	193	4
II.3	130	100	1030	210	2
II.4	96	100	832	201	3

The remaining immune work‐up was normal in patient P2, including blood cell count, assessment of immunoglobulin levels, and serology to protein antigen. Patient P1 exhibited normal T lymphocyte counts (CD4 T cells: 672/µL, reference range: 530–1300/µL; CD8 T cells: 521/µL, reference range: 330–920/µL). However, there was a decreased proportion of naïve CD4 and CD8 T cells (CD4 T cells: 23.7%, reference range: 43–55%; CD8 T cells: 5.9%, reference range: 52–68%), moderate B lymphopenia (B lymphocytes: 168/µL, reference range: 193–628/µL), and normal NK cell counts (NK cells: 228/µL, reference range: 70–480/µL). Moderate lymphopenia and reduced proportions of naïve T cells have been previously documented in patients with DS [12, 13, 14]. The serologies to protein antigens (tetanus and pneumococcus) performed were positive but fell below the thresholds expected for a fully vaccinated patient.

## 4 Discussion

We report two new cases of complete FD deficiency. To date, seven families with complete FD deficiency have been reported. A comprehensive review of all reported cases was conducted, and the findings are summarized in Table 2. Biochemical investigations were performed in all these kindreds but genetic study in only four of them. FD deficiency was first described in 1984 by Kluin‐Nelemans et al. in monozygotic twins presenting with repeated upper and lower respiratory tract infections since childhood caused by *H. influenza, Proteus mirabilis*, and *Pseudomonas aeruginosa* [5]. Since then, twelve patients in six kindreds have been described. Seven of them presented with either recurrent (one patient, *N. meningitidis*, and *N. gonorrhoea*) or severe Neisseria infections (*N. meningitidis*), one with a severe pneumococcal infection and the others were asymptomatic. One of the patients died at 9 months old of meningitis caused by *N. meningitidis*. The median age at the onset of severe or recurrent infections was 7 years old (range: 0–24).

**TABLE 2:** Review of the literature of all reported cases of FD deficiency.

				Clinical features				Biological features					
	Article	Year of publication	Number of cases and relationship	First invasive infection or history of chronic infections at diagnosis	Sex and age at onset	Ethnicity	Outcome and subsequent infections, if available	Pathogens identified		CH50	AP50	FD	Genetics
1	Kluin‐Nelemans HC, et al. Functional deficiency of complement factor D in a monozygous twin. *Clin Exp Immunol*. 1984	1984	2 (Homozygous twins)	Case 1: Recurrent upper and lower respiratory infections Case 2: Recurrent upper and lower respiratory infections	Case 1: Female, 7 years old Case 2: Female; 8 years old	NA	Infections described were recurrent bronchitis, rhinitis, and sinusitis. Case 1 had a resection of the right lower lobe for bronchiectasis.	Case 1: *Hemophilus influenza* *Proteus mirabilis* *Pseudomonas aeruginosa* Case 2: *H. Influenzae*	Case 1 Case 2	278 U/mL –	1.7 (N: 42+ 10.8 U/mL) 8.4 U/mL	4.4 (N: 62 5 + 7.9 U/mL) 4.9 U/mL	NA
2	Hiemstra PS, et al. Complete and partial deficiencies of complement factor D in a Dutch family. *J Clin Invest*. 1989	1989	1	Case 1: Reccurent Neisseria infections	Case 1: Male, 24 years old	NA	Infections described were meningitis, sepsis, and recurrent episodes of fever	Case 1*: N. meningitidis* *Neisseria gonorrhoeae*	Case 1	173 (N: 256–580 U/mL)	<1 (N: 8–24 U/mL)	0 (N: 25–05 U/mL)	NA
3	Weiss SJ, et al. Complement factor D deficiency in an infant first seen with pneumococcal neonatal sepsis. *J Allergy Clin Immunol*. 1998	1998	1	Case 1: Meningitis and sepsis	Case 1: Male, 6 days old	NA	No information available on his evolution	Case 1: *S. pneumoniae*	Case 1	16 (N: 20–40 U/mL)	1 (N: 20–40 U/mL)	Undetectable (radial immunodiffusion)	NA
4	Biesma DH, et al. A family with complement factor D deficiency. *J Clin Invest*. 2001	2001	5: Case 1 is the propositus, Case 2 is her homozygous twins, Case 3 her mother, Case 4 is her uncle, and Case 5 is her great grand cousin	Case 1 (propositus): Meningitis and *pupura fulminans* Cases 2, 3, and 4: No history of repeated or severe infections Case 5: Meningitis	Case 1: Female, 23 years old Case 2: Female, NA Case 3: Female, NA Case 4: Male, NA Case 5: Male, 20 years old	Caucasian	Case 1: Recovered from the infection. Case 2 was asymptomatic at 23 years old. The age of Case 3 and Case 4 are not given but we know they are a generation older than Case 1. Case 5: Died of pneumonia and meningitis at 70 years old.	Case 1: *Neisseria meningitidis* serogroup B (type 15, subtype P1.5) Case 2: NA Case 3: NA Case 4: NA Case 5: First meningitis was undocumented, second and fatal infection: *S. pneumoniae*	Case 1 Case 2 Case 3 Case 4 Case 5	113 (N: 100 ± 25%) 116 116 112 NA	<10 (N: 100± 25%) <10 <10 <10 NA	<0.03 (N: 1.39± 0.32 mg/L) <0.03 <0.03 <0.03 NA	c.125C>A p.Ser42* (Hmz) MAF:
5	Sprong T, et al. Deficient alternative complement pathway activation due to factor D deficiency by 2 novel mutations in the complement factor D gene in a family with meningococcal infections. *Blood*. 2006	2006	2 (siblings)	Case 1: Meningitis with seizures Case 2: Meningitis and petechial rash	Case 1: Female, 9 months old Case 2: Male, 13 months old	Turkish	Case 1: Died of her infection. Case 2: Recovered Both infections did not happen at the same time.	Case 1: *Neisseria meningitidis* serogroup B Case 2: *Neisseria meningitidis* serogroup B	Case 1 Case 2	NA NA	NA <5% (N: 75−125%)	NA <0.03 (1–2 mg/L)	c.638T>C p.Val213Gly (Hmz) MAF: c.640T>C p. Cys214Arg (Hmz) MAF:
6	Sng CCT, et al. A type III complement factor D deficiency: Structural insights for inhibition of the alternative pathway. J *Allergy Clin Immunol*. 2018	2018	2 (siblings)	Case 1 (propositus): Meningitis and petechial rash Case 2: Asymptomatic at diagnosis	Case 1: Female, 19 years old Case 2: Male, NA	South Asian	Case 1: Recovered Case 2 had an undocumented meningitis at 11 years old	Case 1: *Neisseria meningitidis* serogroup Y	Case 1 Case 2	Normal	0% 0%	2.3 mg/L (within normal ranges) 2.0 mg/L	c.602 p.Arg176Pro (Hmz) MAF
7	El Sissy C, et al. Clinical and genetic spectrum of a large cohort with total and subtotal complement deficiencies. *Front Immunol*. 2019	2019	1	Case 1: *Purpura fulminans*	Case 1: Male, 3 years old	NA	Case 1: Recovered	Case 1: *Neisseria meningitidis* serogroup B strain (B:41/44)	Case 1	Normal	<10 (70−130%)	NA	c.677–678delinsTTCT (Htz) MAF c.653T>C p.Leu218Pro (Htz) MAF

Most patients in these cases and patient P2 presented with severe and sometimes recurring meningococcal diseases and some of them were adults [8] when the deficiency was identified. We advocate, similar to other reports [15], for an exploration of the alternative pathway followed by a familial study when needed (including the older members of the family) for all patients with severe meningococcal diseases. This allows for adequate preventive measures including vaccination for encapsulated bacteria and prophylactic antibiotics. Unlike other immunodeficiencies, FD deficiency does not impair a child's development and, in the absence of severe invasive infections, it can remain completely unnoticed until adulthood. Although associated in the reported kindreds with life‐threatening meningococcal, gonococcal, and pneumococcal infections, FD deficiency is likely underdiagnosed due to the absence of systematic immune evaluations in patients presenting with these infections.

Here, we report the case of a 13‐year‐old with Down syndrom (P1) who developed a severe *H. influenzae* infection leading to sepsis, requiring a 39‐day stay in the ICU and resulting in long‐term  sequelae for nearly two years after the infection. Patients with DS usually present with altered lymphocyte counts, imbalance of T‐ and B‐cell subgroups [13, 14], and anatomical abnormalities which altogether lead to increased susceptibility to infections [12]. This may have played a role in the patient's P1 infection. However, decreased activity of the AP has also been associated with severe or recurrent infections to other encapsulated bacteria and we believe the FD deficiency may have had a significant impact on the severity of the patient's P1 infection. We advocate for the evaluation of complement deficiency diseases in patients with Down syndrom who experience severe infections caused by encapsulated bacteria, even in the presence of lymphopenia.

Finally, we emphasize the need for vigilance in patients receiving  FD inhibitors [16, 17, 18, 19, 20]. In paroxysmal noctural hemoglobunria (PNH), therapeutic FD inhibitors were designed to control intravascular hemolysis and prevent C3‐mediated extravascular hemolysis. One of them, danicopan [21], has just been approved as an add‐on therapy to C5 complement inhibitor for the treatment of refractory hemolytic anemia in PNH. Moreover, mannan‐binding lectin‐associated serine protease‐3 (MASP‐3) inhibitors are also being developed. MASP‐3 allows the cleavage of pro‐factor D in FD, thus activating the AP upstream of Factor D. OMS906, a MASP‐3 inhibitor, has shown promising results *in vitro* [22] and in a phase I clinical trial [23]. Several clinical trials on different populations are currently ongoing. Given the severe infections observed in patients with inborn FD deficiency, the infectious risk should be a major concern for clinicians managing patients receiving these treatments. In clinical trials involving FD inhibitors, no severe bacteria‐documented infections were reported [16, 24]. However, patients were vaccinated against encapsulated bacteria as a preventive measure and the only trial with an FD inhibitor in monotherapy was a phase II trial [16] meaning this risk has not yet fully been evaluated in a real‐life setting. Regarding MASP‐3 inhibitors, a phase I study on healthy subjects reported one severe treatment‐emergent adverse event, a tooth abscess. The vaccination status of the enrolled individuals was not disclosed [23]. Therefore, close monitoring of any sign of invasive infections and the implementation of preventive measures against encapsulated bacteria will be essential for the patients receiving FD or MASP‐3 inhibitors.

## Author Contributions

Véronique Frémeaux‐Bacchi, Carine El Sissy, and Paula Vieira Martins: Conceptualization, design, and interpretation of the data. Mathilde Puel, Kenza Rwayane, Paula Vieira Martins, Véronique Frémeaux‐Bacchi, and Carine El Sissy: Acquisition and analysis of the data. Mathilde Puel, Jérémie Rosain, Véronique Frémeaux‐Bacchi, and Carine El Sissy: Drafting the manuscript. All the authors contributed to the writing and critical appraisal of the manuscript.

## Conflicts of Interest

The authors declare no conflicts of interest.

### Peer Review

The peer review history for this article is available at https://publons.com/publon/10.1002/eji.202451536

## Data Availability

The data supporting the findings of this study are available from the corresponding author upon reasonable request.
